# The impact of the histological classification of ampullary carcinomas on long-term outcomes after pancreaticoduodenectomy: a single tertiary referral center evaluation

**DOI:** 10.1007/s00423-022-02563-z

**Published:** 2022-06-07

**Authors:** Giuseppe Quero, Vito Laterza, Claudio Fiorillo, Roberta Menghi, Davide De Sio, Carlo Alberto Schena, Fausto Rosa, Antonio Pio Tortorelli, Ludovica Di Cesare, Caterina Cina, Maria Bensi, Lisa Salvatore, Sergio Alfieri

**Affiliations:** 1 Pancreatic Surgery Unit, Department of Surgery, Gemelli Pancreatic Center, CRMPG (Advanced Pancreatic Research Center), Fondazione Policlinico Universitario “Agostino Gemelli” IRCCS, Largo Agostino Gemelli, 8, 00168 Rome, Italy; 2 Università Cattolica del Sacro Cuore Di Roma, Largo Francesco Vito 1, 00168 Rome, Italy; 3 Comprehensive Cancer Center, Fondazione Policlinico Universitario Agostino Gemelli, IRCCS, Largo Agostino Gemelli, 8, 00168 Rome, Italy

**Keywords:** Ampullary carcinoma, Long-term outcomes, Histotype, Overall survival, Disease-free survival

## Abstract

**Purpose:**

Ampullary carcinomas (ACs) are classified as pancreatobiliary (Pb-AC), intestinal (Int-AC), or mixed (Mixed-AC). The influencing role of AC subtypes on long-term outcomes is still matter of debate. Aim of this study is to evaluate the prognostic role of the three histological variants on the overall (OS) and disease-free survival (DFS) after pancreaticoduodenectomy(PD).

**Methods:**

All PDs for AC between 2004 and 2020 were included. Patients were classified according to the histological feature in Pb-AC, Int-AC, and Mixed-AC. Five-year OS and DFS were compared among the subtypes. Additionally, the prognostic role of the histological classification on OS and DFS was evaluated.

**Results:**

Fifty-six (48.7%) Pb-ACs, 53 (46.1%) Int-ACs, and 6 (5.2%) Mixed-ACs were evaluated. A poorer 5-year OS was evidenced for the Pb-AC group (54.1%) as compared to the Int-AC cohort (80.7%) (*p* = 0.03), but similar to the Mixed-AC population (33%) (*p* = 0.45). Pb-AC presented a worse 5-year DFS (42.3%) in comparison to the Int-AC (74.8%) (*p* = 0.002), while no difference was evidenced in comparison to the Mixed-AC (16.7%) (*p* = 0.51). At the multivariate analysis, the Pb-/Mixed-AC histotype was recognized as negative prognostic factor for both OS (OR: 2.29, CI: 1.05–4.98; *p* = 0.04) and DFS (OR: 2.17, CI: 1–4.33; *p* = 0.02).

**Conclusion:**

Histological subtypes of AC play a relevant role in long-term outcomes after PD. Pb-ACs and Mixed-ACs show a more aggressive tumor biology and a consequent worse survival as compared to the Int-AC subtype.

## Introduction

Ampullary carcinomas (ACs) are rare neoplasms arising from the papilla of Vater, and currently represent the 7% of periampullary tumors and approximately the 0.2% of all gastrointestinal neoplasms [1]. Histologically, the papilla of Vater is composed of pancreatobiliary and intestinal epithelium either of which can give origin to cancer. This leads, since 1994, to the recognition of two different histological patterns of AC: the intestinal and the pancreatobiliary variants [2]. A more recent AC histological classification recognizes also a third histological subtype (Mixed-AC), with intermediate histological features between the intestinal and pancreatobiliary histotypes [3]. The intestinal type arises from the intestinal mucosa located at the papilla, subsequently evolving through an adenoma-carcinoma sequence, similarly to colorectal and duodenal malignancies [4]. On the counterpart, the pancreatobiliary type originates from the epithelium of the ampulla, the distal common bile duct, or the distal pancreatic duct, subsequently evolving from precursor pancreatic intraepithelial neoplasia, similarly to pancreatic adenocarcinoma [4]. Conversely, the mixed histotype is a tumor that consists of more than 25% of each differentiation [5].

In terms of oncological outcomes, AC is characterized by a more favorable course in comparison to other periampullary malignancies, with a reported 5-year survival rate of almost 45% in resected patients as compared to 20–30% and 15–20% in case of the distal common bile duct and pancreatic head carcinomas, respectively [1, 6]. Although disease stage, lymph node involvement, and tumor grading have been widely recognized as the main influencing features on the prognosis of patients affected by AC [1, 3, 7–10], the influencing role of the histopathological characteristics of ACs on oncological outcomes is still a matter of debate. According to some authors, the pancreaticobiliary variant correlates to a higher tumor stage, greater risk of lymph node metastasis, and greater tendency towards perineural invasion, leading to a significantly poorer disease-free survival (DFS) and overall survival (OS) as compared to the intestinal type [11–15]. On the counterpart, other authors did not find any significant difference between the two histotypes in terms of oncological outcomes [16–18].

These contrasting data may find justification in the low incidence of ACs and the consequent small cohorts of patients involved in the analyses, thus limiting the reliability of the results.

Given these premises, the aim of the present study is to compare the clinico-pathological characteristics and the long-term outcomes (in terms of DFS and OS) among the pancreatobiliary, intestinal, and mixed types of AC after curative-intent resection in a tertiary referral center for the treatment of periampullary malignancies.

## Material and methods

After the Institutional Review Board (IRB) approval, all patients who underwent pancreaticoduodenectomy (PD) for a histologically proven diagnosis of AC at the Pancreatic Surgery Center of the Fondazione Policlinico “Agostino Gemelli” IRCCS of Rome from January 2004 to December 2020 were retrospectively included in the study.

Patients were classified according to the AC histopathological pattern into the pancreaticobiliary histotype (Pb-AC), intestinal histotype (Int-AC), and mixed histotype (Mixed-AC) groups.

All PDs were performed by senior pancreatic surgeons and all patients underwent a Whipple procedure with standard lymphadenectomy [19, 20]. Specifically, lymph node stations 5, 6, 8a, 12b1, 12b2, 12c, 13a, 13b, 14a, 14b, and 17a were dissected in all cases.

Perioperative data collected were clinico-demographic characteristics (sex and age) and intraoperative (operative time and blood loss) and post-operative data (complications, length of hospital stay, 30-day mortality, adjuvant therapy).

Post-operative complications were defined as any deviation from the post-operative course and classified according to the Clavien-Dindo classification [21], while 30-day mortality was defined as any death that occurred within 30 days after surgery.

Post-operative pancreatic fistula was defined and graded according to the International Study Group of Pancreatic Surgery (ISGPS) criteria [22].

Histopathological features of analysis included tumor dimension and grading, number of harvested lymph nodes and number of positive lymph nodes, evaluation of lymphovascular and perineural invasion, TNM staging according to the 8^th^ edition of the AJCC/UICC system, and resection margin status. The microscopic positivity of resection margins (R1) was defined as the detection of tumor cells within 1 mm from the transection margin, according to the Royal College of Pathologists [23]. Although the classification of the histological subtypes was reported at histology after surgery in all cases, all the histopathological slides were retrospectively reviewed by expert pancreatic pathologists, furtherly confirming the classification into Pb-, Int-, and Mixed-AC subtypes according to the World Health Organization (WHO) 2019 classification [24]. Moreover, all ACs were retrospectively re-classified for staging according to the 8^th^ edition of the AJCC/UICC system.

With regard to the employment of post-operative chemotherapy, adjuvant treatment was prescribed in the case of advanced T stages (T3 tumors), lymph node metastases, and positive resection margins.

### Post-operative follow-up and long-term outcome evaluation

Post-operative follow-up was previously reported [19, 25]. In brief, patients underwent physical examination, laboratory tests (here including oncological markers), and transabdominal ultrasound every 3 months for the first 2 years after PD. A magnetic resonance imaging (MRI) and/or computed tomography (CT) was prescribed annually and anticipated in case of suspicion of tumor recurrence at the transabdominal ultrasound or in case of elevated tumor markers. A whole-body positron emission tomography (PET) scan with 18-fluoro-2-deoxy-glucose (FDG) was performed in case of an inconclusive diagnosis at the CT and/or MRI.

Analysis of long-term outcomes included recurrence rate, OS, and DFS. Local recurrence was defined as the retroperitoneum tumor relapse at the pancreatic remnant, regional lymph nodes, and around the mesenteric and/or celiac vessels, while distant metastases were defined as tumor relapse at any other site, here including peritoneal dissemination and liver and para-aortic lymph node metastases. OS was defined as the time from surgery to the last follow-up, while DFS was defined as the time from surgery to the first evidence of tumor recurrence or death.

### Study outcomes

The primary aim of the study was a comparison among the Pb-AC, Int-AC, and Mixed-AC groups in terms of OS. Secondary aims were a further comparison among the three study groups in terms of tumor recurrence (local or distant), DFS, and perioperative outcomes.

### Statistical analysis

Continuous data were reported as means ± standard deviations (± SDs), while numbers and percentages were used for categorical variables. Student’s *t* tests, Mann–Whitney *U* tests, Fisher’s tests, and *χ*^2^ tests were used for the univariate analysis. A *p* value ≤ 0.05 was considered statistically significant. OS and DFS were calculated using the Kaplan–Meier method, and the Cox proportional hazard model was used for multivariable analysis. Results were reported as odds ratio (OR) with 95% confidence intervals (CI). A two-tailed *p* value < 0.05 was considered statistically significant. All tests were performed using SPSS version 25 for Windows (SPSS Inc., Chicago, IL, USA).

## Results

From January 2004 to December 2020, a total of 574 PDs were performed at the Pancreatic Surgery Center of the Fondazione Policlinico Universitario Agostino Gemelli IRCCS of Rome. One-hundred and fifteen patients (20%) were diagnosed with AC, and, thus, included in the study.

At the histopathological examination, 56 patients (48.7%) presented a pancreatobiliary histotype (Pb-AC group), 53 (46.1%) an intestinal histotype (Int-AC group), while the mixed histotype (Mixed-AC group) was identified in the remaining 6 patients (5.2%).

Table 1 shows the clinico-demographic and pathological characteristics of the three subtype groups. The three populations were comparable for demographic data and intraoperative outcomes. Similarly, no difference was evidenced among the three cohorts in terms of post-operative development of complications, length of hospital stay, and 30-day mortality.

**Table 1:** Clinico-demographic characteristics and intra- and post-operative outcomes of the three study cohorts

	Pb-AC (*n* = 56)	Int-AC (*n* = 53)	Mixed-AC (*n* = 6)	*p*
**Preoperative data**				
Sex, *n* (%)				
Male	17 (30.4)	23 (43.4)	3 (50)	0.3
Female	39 (69.6)	30 (56.6)	3 (50)	
Age (years), *mean (*± *SD)*	65.6 (± 10.2)	64.5 (± 11.5)	68 (7.7)	0.92
ASA score, *n (%)*				
I	6 (10.7)	8 (15.1)	1 (16.7)	0.76
II	41 (73.2)	39 (73.6)	5 (83.3)	
III	9 (16.1)	6 (11.3)	0	
**Intraoperative outcomes**				
Operative time (min), *mean (*± *SD)*	310.3 (± 81.3)	298.4 (± 53.1)	321.6 (± 78.6)	0.15
EBL (mL), *mean (*± *SD)*	201.7 (± 98.4)	270.4 (± 108.3)	248 (± 76.5)	0.21
**Post-operative outcomes**				
Post-operative complications, *n (%)*				0.75
Clavien-Dindo I–II	16 (28.6)	14 (26.4)	3 (50)	
Clavien-Dindo III–IV	9 (16)	7 (13.2)	1 (16.7)	
POPF, *n (%)*				
Biochemical leak	17 (30.3)	16 (30.2)	2 (33.3)	0.97
Grade B	10 (17.8)	11 (20.7)	1 (16.7)	
Grade C	1 (1.8)	0	0	
Length of hospital stay (days), *mean (*± *SD)*	16.1 (± 9.1)	17.4 (± 11.4)	12.5 (± 8.3)	0.2
30-day mortality, *n (%)*	2 (3.6)	2 (3.7)	0	0.89

*ASA* American Society of Anesthesiologists; *EBL* estimated blood loss; *POPF* post-operative pancreatic fistula

At the histopathological evaluation (Table 2), Pb-ACs more frequently presented an advanced T stage [37 (66.1%) T3 tumors] as compared to Int-ACs [18 (34%)] and Mixed-ACs [2 (33.3%)] (*p* = 0.003). Similarly, the Pb- and Mixed-ACs presented a higher frequency of lymph node involvement (N +) (60.7% and 50%, respectively) than the Int-AC group (34%) (*p* = 0.02). Furthermore, the Pb-AC and Mixed-AC histotypes were significantly associated with a higher rate of perineural and lymphovascular invasion in comparison to the Int-AC group (*p* = 0.02 and *p* = 0.05, respectively). Conversely, the three histotypes were comparable for number of harvested lymph nodes (*p* = 0.07), mean number of positive lymph nodes (*p* = 0.2), and R status (*p* = 0.78).

**Table 2:** Histopathological features of the study populations

	Pb-AC (*n* = 56)	Int-AC (*n* = 53)	Mixed-AC (*n* = 6)	*p*
**Histopathological data**				
Tumor size (cm), *mean (*± *SD)*	2.2 (± 1)	2.2 (± 1.2)	2 (± 0.6)	0.91
T stage				
T1	6 (10.7)	10 (18.9)	0	**0.003**
T2	13 (23.2)	25 (47.1)	4 (66.7)	
T3	37 (66.1)	18 (34)	2 (33.3)	
N + , *n (%)*	34 (60.7)	18 (34)	3 (50)	**0.02**
Harvested lymph nodes, *mean (*± *SD)*	17 (± 7.5)	14.4 (± 8.4)	15.2 (± 4.2)	0.07
Positive lymph nodes, *mean (*± *SD)*	1.27 (± 1.9)	0.8 (± 1.7)	0.8 (± 1.17)	0.2
Perineural invasion, *n (%)*	33 (58.9)	20 (37.7)	5 (83.3)	**0.02**
Lymphovascular invasion, *n (%)*	39 (69.6)	25 (47.2)	4 (66.7)	**0.05**
Tumor grading, *n (%)*				
G1	2 (3.6)	6 (11.3)	0	0.06
G2	34 (60.7)	37 (69.8)	6 (100)	
G3	20 (35.7)	10 (18.9)	0	
R1, *n (%)*	4 (7.1)	4 (7.5)	0	0.78

*R1*microscopic positivity of resection margins

The mean follow-up of the entire cohort was 60.2 (± 5.6) months (Table 3). Sixty-seven patients (58.2%) received an adjuvant treatment, with a higher frequency in the Pb-AC and Mixed-AC groups [42 (75%) and 3 (50%), respectively] in comparison to the Int-AC cohort [22 (41.5%)] (*p* = 0.002). Adjuvant regimens did not vary over the study period: gemcitabine-based regimens (gemcitabine or gemcitabine + cisplatin) were employed in all cases of Pb- and Mixed-AC, while fluoropyrimidine-based regimens (capecitabine/DEGRAMONT or FOLFOX) were used for Int-ACs, for a treatment duration of 6 months. As a whole, the recurrence rate was 46% (53 patients), with a higher frequency for the Pb-AC and Mixed-AC histotypes as compared to the Int-AC variant (*p* = 0.003). Local tumor relapse was documented in 18 patients (34%), while distant recurrence was observed in 31 cases (58.5%), while the remaining 4 patients (7.5%) presented both local and distant recurrence.

**Table 3:** Follow-up outcomes

	Pb-AC (*n* = 56)	Int-AC (*n* = 53)	Mixed-AC (*n* = 6)	*p*
Follow-up, *mean (*± *SD)*	62.4	58.3	60.1	0.1
Adjuvant therapy, *n (%)*	42 (75)	22 (41.5)	3 (50)	**0.002**
Recurrence rate, *n (%)*	32 (57.1)	16 (30.2)	5 (83.3)	**0.003**

**Fig. 1:**
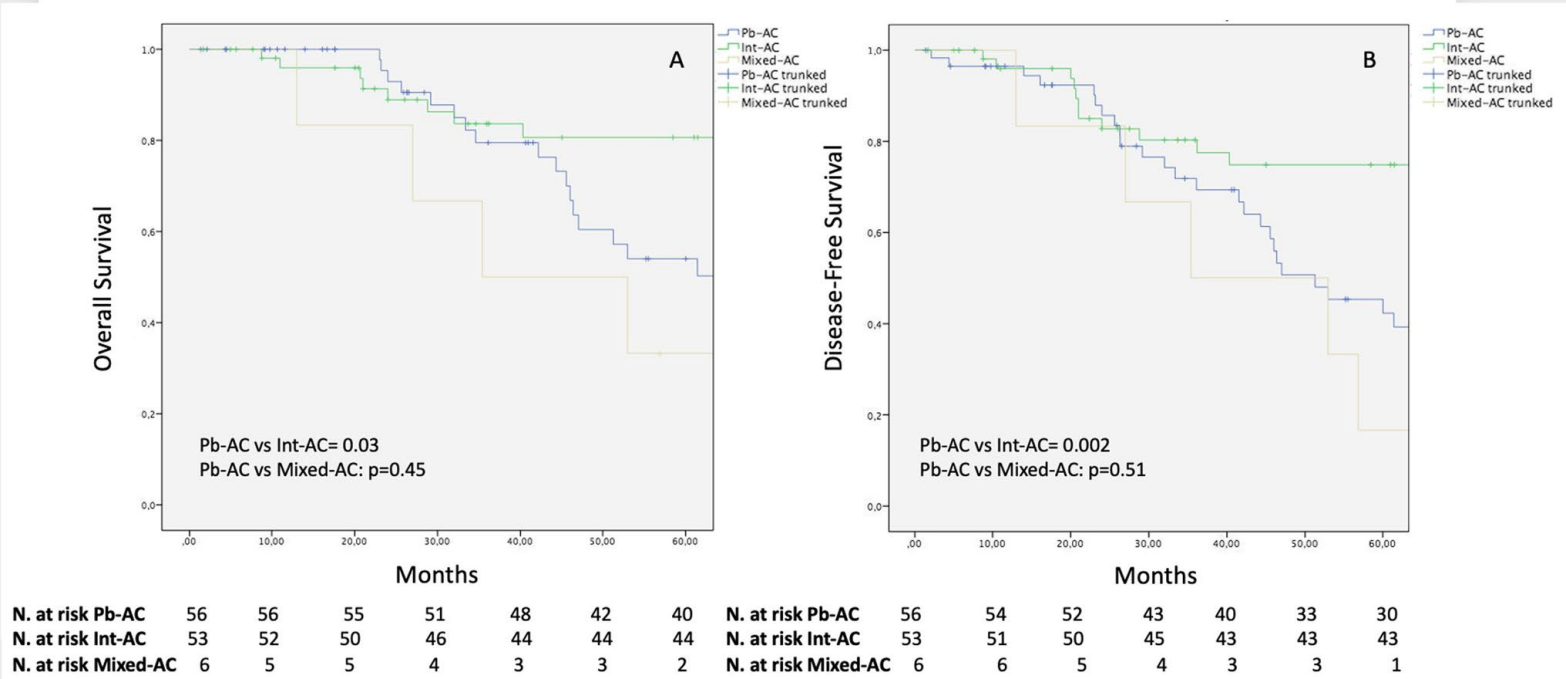
Kaplan–Meier curves comparing OS (**A**) and DFS (**B**) among the Pb-AC, Int-AC, and Mixed-AC cohorts

**Fig. 2:**
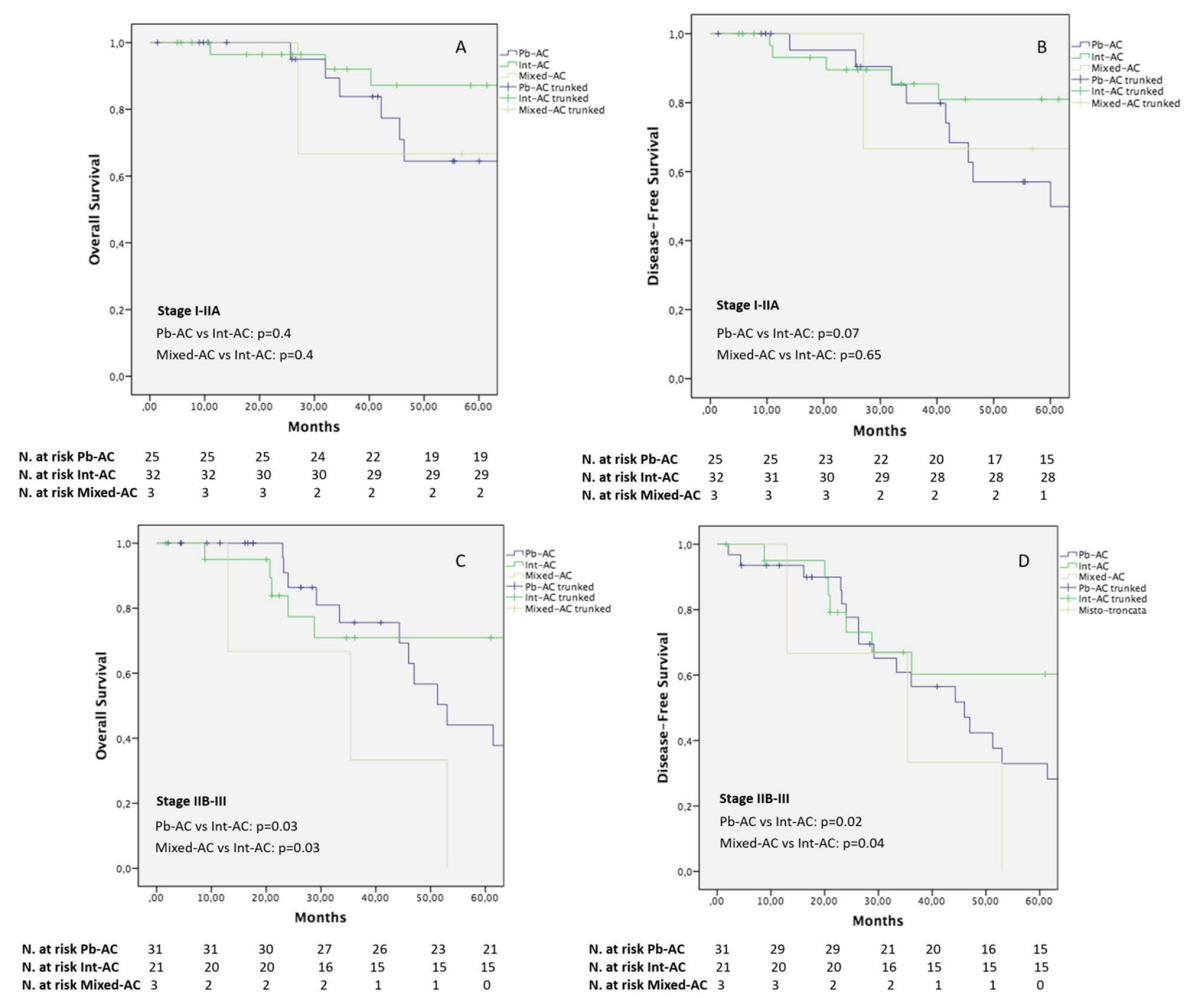
Kaplan–Meier curves comparing OS and DFS among the Pb-AC, Int-AC, and Mixed-AC cohorts according to stages I–IIA (**A**–B) and IIB–III (**C**–**D**)

The 5-year DFS and OS curves are reported in Fig. 1 A–B. One-, 3-, and 5-year DFS rates were significantly lower for the Pb-AC histotype (96.4%, 71.8%,and 42.3%, respectively) in comparison to the Int-AC variant (95.9%, 80.3%, and 74.8%, respectively; *p* = 0.002), while no difference was evidenced as compared to the Mixed-AC histotype (100%, 50%, and 16.7%, respectively; *p* = 0.51). Similarly, 1-, 3-, and 5-year OS rates of the Pb-AC groups (100%, 79.5%, and 54.1%, respectively) were significantly lower as compared to the Int-AC population (100%, 83.6%, and 80.7%, respectively; *p* = 0.03) but similar to the Mixed-AC cohort (100%, 50%, and 33.3%, respectively; *p* = 0.45). When stratified for disease stage (Fig. 2), no difference was noted among the three subtypes in terms of OS and DFS at early stages (Fig. 2A and B). Conversely, a significantly better OS was evidenced for stages IIB–III Int-ACs in comparison to the Pb-ACs (*p* = 0.03) and Mixed-ACs (*p* = 0.03). Similarly, a better DFS was documented at advanced stages for Int-ACs as compared to the pancreatobiliary (*p* = 0.02) and mixed histotypes (*p* = 0.04) (Fig. 2C and D).

**Fig. 3:**
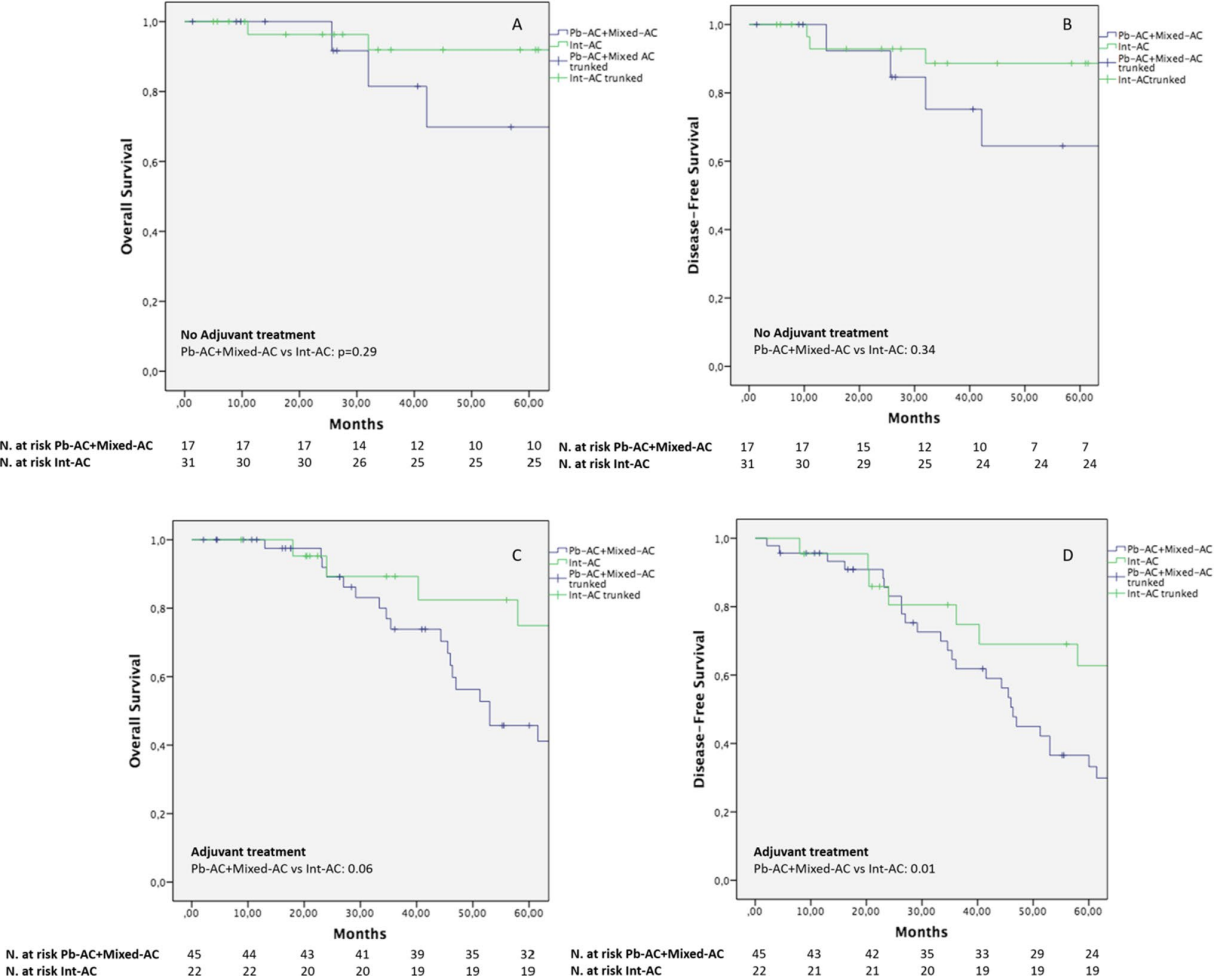
Kaplan–Meier curves comparing OS and DFS between Pb- + Mixed-AC and Int-AC in case of no adjuvant (**A**–**B**) and adjuvant treatment (**C**–**D**)

### Predictive factors analysis for OS and DFS (Table 4)

**Table 4:** Univariate and multivariate analysis for OS and DFS

Variable	Univariate analysis				Multivariate analysis					
	5-year OS (%)	*p*	5-year DFS (%)	*p*	5-year OS			5-year DFS		
					OR	95% CI	*p*	OR	95% CI	*p*
Age < 65/ ≥ 65	68.7/62.9	0.1	61.8/53.3	0.23	-	-	-	-	-	-
Sex, M/F	73.6/58.9	0.56	68.8/49.3	0.37	-	-	-	-	-	-
ASA, I–II/III	68.5/53.3	0.42	57.6/53.3	0.11	-	-	-	-	-	-
EBL, ≤ 225/ > 225 mL^a^	59.3/72.5	0.6	58.3/71.4	0.68	-	-	-	-	-	-
Complications, yes/no	55.8/74.4	0.06	62.8/53.8	0.37	-	-	-	-	-	-
Histotype, Pb-Mixed/Int	51.3/80.7	**0.001**	41.1/74.8	**0.001**	2.29	1.05–4.98	**0.04**	2.17	1–4.33	**0.02**
T stage, 1–2/3	78.2/54.3	**0.004**	74.5/43.9	** < 0.0001**	2.7	1.2–6	**0.01**	1.65	0.81–3.35	0.16
Tumor grading, 1–2/3	65.9/52.3	0.44	57.3/56.7	0.83	-	–	–	–	–	-
Lymph nodes harvested, ≤ 15/ > 15^a^	52.8/72.9	**0.04**	48.1/63.3	**0.04**	1.82	0.9–3.69	0.09	1.46	0.77–2.8	0.24
Positive lymph nodes, yes/no	76.6/46.7	** < 0.0001**	37/71.3	** < 0.0001**	2.8	1.43–5.45	**0.002**	2.8	1.48–5.29	**0.001**
Perineural invasion, yes/no	71.7/58.8	0.06	48.9/69.1	**0.003**	-	–	–	1.32	0.68–2.56	0.4
Lymphovascular invasion, yes/no	87.3/51.4	**0.01**	44.7/77.1	**0.01**	2.9	1.3–6.7	**0.002**	1.5	0.77–2.89	0.23
R status, positive/negative	25/67.2	**0.03**	20/59.6	**0.01**	1.2	0.34–4.17	0.77	1.63	0.6–4.4	0.33
Adjuvant therapy, yes/no	61.7/67.7	0.34	62.3/48.5	0.12	-	-	-	-	-	-

^a^The mean number of EBL and lymph nodes retrieved was used as cutoff for the univariate and multivariate analyses

*ASA* American Society of Anesthesiologists; *EBL* estimated blood loss; *R* resection margin

At the univariate analysis, OS was significantly influenced by the Pb-Mixed-AC histotype (*p* = 0.001), T3 staging (*p* = 0.004), more than 15 lymph nodes harvested (*p* = 0.04), metastatic lymph nodes (*p* < 0.0001), lymphovascular invasion (*p* = 0.01), and positive resection margins (*p* = 0.03). However, at the multivariate analysis, only the Pb-Mixed-AC histotype (OR: 2.29, CI: 1.05–4.98; *p* = 0.04), the T3 stage (OR: 2.7, CI: 1.2–6; *p* = 0.01), the presence of metastatic lymph nodes (OR: 2.8, CI: 1.43–5.45; *p* = 0.002), and lymphovascular invasion (OR: 2.9, CI: 1.3–6.7; *p* = 0.002) were recognized as independent influencing factors on OS.

Similarly, at the univariate analysis, the Pb-Mixed-AC histotype (*p* = 0.001), T3 stage (*p* < 0.0001), more than 15 lymph nodes harvested (*p* = 0.04), metastatic lymph nodes (*p* < 0.0001), perineural and lymphovascular invasion (*p* = 0.003 and *p* = 0.01, respectively), and resection margin positivity (*p* = 0.01) were recognized as potential prognostic features for DFS. Of these, only the Pb-Mixed-AC histotype (OR: 2.17, CI: 1–4.33; *p* = 0.02) and lymph node metastases (OR: 2.8, CI: 1.48–5.29; *p* = 0.001) were confirmed as negative prognostic factors for DFS.

## Discussion

The recent demonstration of a better overall prognosis for AC in comparison to pancreatic ductal adenocarcinoma (PDAC) [1, 6] has led to a growing interest towards a deeper understanding of ampullary malignancies behavior, and the hypothesis that differences in AC histology may have relevant clinical implications on long-term outcomes has become one of the main focus of research.

In order to give our contribution to this topic, we investigated the long-term prognostic value of the histological classification of AC in one of the largest patients’ cohorts (*n* = 115) reported in the current literature. From the analysis we conducted, three main findings should be outlined. First, both the Pb-AC and Mixed-AC histotypes demonstrated a more aggressive biological behavior as compared to the Int-AC variant, being associated with a more advanced tumor stage (*p* = 0.003) and a higher rate of perineural (*p* = 0.02) and lymphovascular invasion (*p* = 0.05). Second, at the survival analysis, the more aggressive feature of Pb-ACs and Mixed-ACs reflected in poorer long-term outcomes, both in terms of 5-year DFS and OS, as compared to Int-AC. Third, we found AC histotype to be an independent prognostic factor at the multivariate analysis for both OS (OR: 2.29, CI: 1.05–4.98; *p* = 0.04) and DFS (OR: 2.17, CI: 1–4.33; *p* = 0.02).

The major pathological “aggressiveness” of the Pb- and Mixed-ACs (as demonstrated by more advanced tumor staging, a higher frequency of lymph node involvement, and perineural and lymphovascular invasion) in comparison to the intestinal variant is consistent with previous reports [11, 13]. This diverse tumor behavior may find justification in the different origins of the neoplastic epithelium of the three subtypes. Specifically, Pb-AC arises from the epithelium of the common bile duct, pancreatic duct, or common ampullary duct and is characterized by a disseminating growth and desmoplastic stromal reaction [13]. On the other hand, Int-AC originates from the intestinal mucosa covering the papilla, and its milder biological behavior could be explained by the existence of precursor adenomas following the adenoma-dysplasia-adenocarcinoma sequence [13, 26, 27]. The Mixed-AC differentiation, conversely, presents intermediate characteristics between the two previous subtypes, showing an intestinal architecture with pancreatobiliary citology [5]. In this last regard, some authors speculated that pancreaticobiliary characteristics are prevalent over intestinal features, potentially driving a more aggressive behavior in comparison to the Int-AC [28].

As a consequence of the less favorable histological features of the Pb- and Mixed-AC histotypes, we found a significantly higher rate of lymph node metastases in these two subgroups of patients (60.7% and 50%, respectively) as compared to the intestinal variant cohort (34%) (*p* = 0.02), further confirming the results already reported by Zimmermann et al. [13] (64.7% vs 25% of N + for the Pb- and Int-ACs, respectively; *p* > 0.001). Conversely, a similar rate of positive resection margins (R1) was documented in the three cohorts (*p* = 0.78), with an incidence rate of 7% in the whole population (8 out of 115 patients). This finding is in line with previous experiences [13, 29–32] that documented an R1 rate ranging between 4 and 16% after pancreaticoduodenectomy for ampullary carcinomas.

Although the majority of authors recognized more aggressive pathological features for the Pb-ACs in comparison to Int-ACs [14], survival evaluations of the different histotypes currently show contrasting results [11–18]. Retrospective studies with consistent cohorts of patients [13, 28] and a more recent meta-analysis [14] reported a significantly poorer 5-year survival rate for Pb-AC as compared to Int-AC. Conversely, some authors found no survival advantage for Int-AC [16–18], despite the presence of more advanced histopathological features for the Pb-AC [16]. From the long-term outcome analysis we conducted, the less favorable pathological characteristics of Pb-AC and Mixed-AC reflected in significantly worse OS and DFS as compared to the intestinal subtype (Fig. 1). This is particularly due to a worse long-term clinical course in the case of advanced stages Pb-/Mixed-ACs, further confirming the major aggressiveness of the Pb-/Mixed-AC histotype in comparison to the intestinal one (Fig. 3).

Based on these preliminary data, in order to further investigate and confirm the potential role that AC histotype plays on long-term outcomes, we also conducted a multivariate analysis for both OS and DFS.

Interestingly, the Pb-/Mixed-AC histotype was confirmed as an independent prognostic factor for both OS and DFS, with an OR of 2.29 and 2.17, respectively. These data are in line with the previous experience reported by Williams et al. [33] who documented an approximately twofold higher risk of worse survival in the case of Pb-AC as compared to the intestinal subtype. This same conclusion was also reached by Zimmerman et al. [13] in their cohort of analysis of 119 ACs. As several other studies on prognostic factors for ACs [30, 34–36], we further confirmed the presence of lymph node metastases, T stage, and lymphovascular invasion as additional independent predictors of poor OS and/or DFS. Surprisingly, resection margin positivity was confirmed as a negative prognostic factor for OS and DFS only at the univariate analysis, while a no statically significant value was documented at the Cox regression analysis. This data, in evident contrast with the current reports in the literature [30], should be taken with caution, since the number of resection margin positivity in our cohort (*n* = *8*) is too low to draw solid conclusions.

Finally, our results showed a statistically significant difference in administering adjuvant therapy, with 75% of patients with pancreaticobiliary subtype as compared with 41.5% and 50% for intestinal and mixed type, respectively (*p* = 0.002). This difference was probably determined by more aggressive histopathological features of Pb-AC. However, at the multivariate analysis, adjuvant therapy itself was not recognized as an influencing factor on both 5-year OS and DFS. For instance, the use of adjuvant regimens after PD for ACs is still a matter of debate, and no consensus is currently present in the literature on its potential long-term benefits [28]. Although some retrospective studies reported a potential long-term benefit from adjuvant chemotherapy in the case of AC with lymph node involvement [37–39], other authors [37] found no difference in terms of survival in the subgroup analysis of patients with different histotypes of AC after administration of gemcitabine or 5-FU versus observation. Given the inconclusive current evidence, it is implicit that prospective clinical trials are still needed in order to better evaluate the role of adjuvant therapy in AC patients.

Our study presents some limitations. First, its retrospective design could have led to possible selection bias. Secondly, due to the low incidence of AC, the sample size may be insufficient to draw definitive conclusions, although, as stated before, our study represents one of the largest cohorts in the current literature. Moreover, compared with other studies which included in the analysis other malignancies of the periampullary region, we attempted to minimize the selection bias of our cohort focusing only on AC.

## Conclusion

In conclusion, the present study has shown that the histopathological classification of AC has a major impact on long-term prognosis, together with other well-known factors such as T staging and lymph node involvement. In particular, the Pb-AC is associated with a poorer prognosis as compared to Int-AC, due to a more aggressive behavior in terms of T staging and lymphovascular and perineural invasion. Furthermore, even if rare, the Mixed-AC histotype appears to show similar pathological behavior to the Pb-AC variant.

Based on these findings, we believe that reporting the histopathologic subtype of AC should be mandatory and considered an independent prognostic factor for long-term outcomes. The conduction, in the near future, of prospective studies to define the effects of subtype-stratified post-operative chemotherapy regimens is needed in order to select tailored treatments for patients affected by AC.
